# SMMDA: Predicting miRNA-Disease Associations by Incorporating Multiple Similarity Profiles and a Novel Disease Representation

**DOI:** 10.3390/biology11050777

**Published:** 2022-05-20

**Authors:** Bo-Ya Ji, Liang-Rui Pan, Ji-Ren Zhou, Zhu-Hong You, Shao-Liang Peng

**Affiliations:** 1 College of Computer Science and Electronic Engineering, Hunan University, Changsha 410200, China; byj@hnu.edu.cn (B.-Y.J.); lip141772@gmail.com (L.-R.P.); 2 College of Computer Science, Northwestern Polytechnic University, Xi’an 710072, China; zhoujiren@mail.nwpu.edu.cn

**Keywords:** miRNA-disease associations prediction, deep neural network, ensemble learning, XGBoost

## Abstract

**Simple Summary:**

Predicting possible associations between miRNAs and diseases would provide new perspectives on disease diagnosis, pathogenesis, and gene therapy. In this work, considering the limited accessibility, high time consumption and high cost in traditional biological researches, we presented a novel computational method called SMMDA by incorporating multiple similarity profiles and a novel disease rep-resentation to accelerate the identification of potential miRNA-disease associations. SMMDA was intended to be useful for the prediction of associations between miRNAs and diseases, and to be effective for prevention, diagnosis, treatment and prognosis of Human diseases.

**Abstract:**

Increasing evidence has suggested that microRNAs (miRNAs) are significant in research on human diseases. Predicting possible associations between miRNAs and diseases would provide new perspectives on disease diagnosis, pathogenesis, and gene therapy. However, considering the intrinsic time-consuming and expensive cost of traditional Vitro studies, there is an urgent need for a computational approach that would allow researchers to identify potential associations between miRNAs and diseases for further research. In this paper, we presented a novel computational method called SMMDA to predict potential miRNA-disease associations. In particular, SMMDA first utilized a new disease representation method (MeSHHeading2vec) based on the network embedding algorithm and then fused it with Gaussian interaction profile kernel similarity information of miRNAs and diseases, disease semantic similarity, and miRNA functional similarity. Secondly, SMMDA utilized a deep auto-coder network to transform the original features further to achieve a better feature representation. Finally, the ensemble learning model, XGBoost, was used as the underlying training and prediction method for SMMDA. In the results, SMMDA acquired a mean accuracy of 86.68% with a standard deviation of 0.42% and a mean AUC of 94.07% with a standard deviation of 0.23%, outperforming many previous works. Moreover, we also compared the predictive ability of SMMDA with different classifiers and different feature descriptors. In the case studies of three common Human diseases, the top 50 candidate miRNAs have 47 (esophageal neoplasms), 48 (breast neoplasms), and 48 (colon neoplasms) are successfully verified by two other databases. The experimental results proved that SMMDA has a reliable prediction ability in predicting potential miRNA-disease associations. Therefore, it is anticipated that SMMDA could be an effective tool for biomedical researchers.

## 1. Introduction

MicroRNAs (miRNAs) constitute a group of about 22 nucleotide long noncoding RNAs, prevalent in flora and fauna [1]. It acts as an essential regulatory factor of gene expressions that participate in degradation or post-transcriptional repression by supplementarily binding to corresponding 3′untranslated regions of their mRNA [2].

By targeting multiple transcripts, miRNAs play pivotal roles in biological processes, such as cell development [3,4,5], apoptosis [6], metabolism [7] and so on. Recently, an increasing amount of researches have revealed the effectiveness of microRNAs as prognostic biomarkers or important diagnostic and promising therapeutic targets for the treatment of malignant tumors [8]. The expression of hsa-miR-17-3p is altered in lung cancer from smokers and the methylation levels of hsa-miR-124-2 were reduced in SiHa cells [9]. The critical role of miRNAs in humans has attracted the attention of many researchers, and traditional in vitro experimental methods have been used to investigate the association between miRNAs and human diseases, and many significant results have been achieved. However, biological in vitro experiments require high human and financial costs and are not destined to study large-scale miRNA and disease data. In recent years, machine learning, deep learning, and other methods have improved and integrated bioinformatics problems. Accordingly, more and more researchers are trying to use methods such as machine learning to conduct miRNA-human disease studies.

Based on the hypothesis that interacting miRNA-disease pairs are more functionally similar and tend to be associated with the same miRNAs or diseases [10,11,12], computational models for predicting miRNA–disease associations have emerged in recent years. For example, Chen et al. [13] developed a heterogeneous label propagation method (HLPMDA) by propagating a heterogeneous label in the multiple networks of miRNAs, diseases, and lncRNAs to predict miRNA-disease associations. Ji et al. [10] focused on constructing a human biological association network using the association between miRNAs and diseases, and other biomolecules in the human body for predicting potential associations between miRNAs and diseases. In addition, this work also introduces graph representation learning methods and deep stacked autoencoder methods to obtain excellent prediction performance. Chen et al. [14] invented a bipartite network projection method (BNPMDA) by fusing integrated miRNA and disease similarity to predict miRNA-disease associations. In this work, a bipartite network recommendation method was applied to predict the potential associations between miRNAs and diseases.

In addition, machine learning approaches have been widely investigated in bioinformatics for predicting potential associations between miRNAs and diseases [15]. For example, Ji et al. [16] used a typical integrated learning approach, random forest, for the potential association of miRNAs with human diseases. They designed an attribute network embedding approach to construct a model with mighty predictive power by considering both the attribute features and network features using a typical integrated learning approach, random forest, for the potential association of miRNAs with human diseases. Zheng et al. utilized deep auto-encoder neural network (AE) and random forest classifier to predict potential miRNA-disease associations (MLMDA). Xu et al. [17] proposed a novel-method-based miRNA target–dysregulated network. Based on the changes and features in miRNA expression, they used SVM classifier to general predictive accuracy. Zhang et al. [18] utilized a variational auto-encoder approach for miRNA-disease association prediction, called VAEMDA. They constructed two spliced matrices by combining the integrated miRNA similarity and the integrated disease similarity with known miRNA–disease associations, respectively. This method prevents the noise created by the random selection of negative instances and shows miRNA-disease associations from the viewpoint of data distribution.

**Figure 1:**
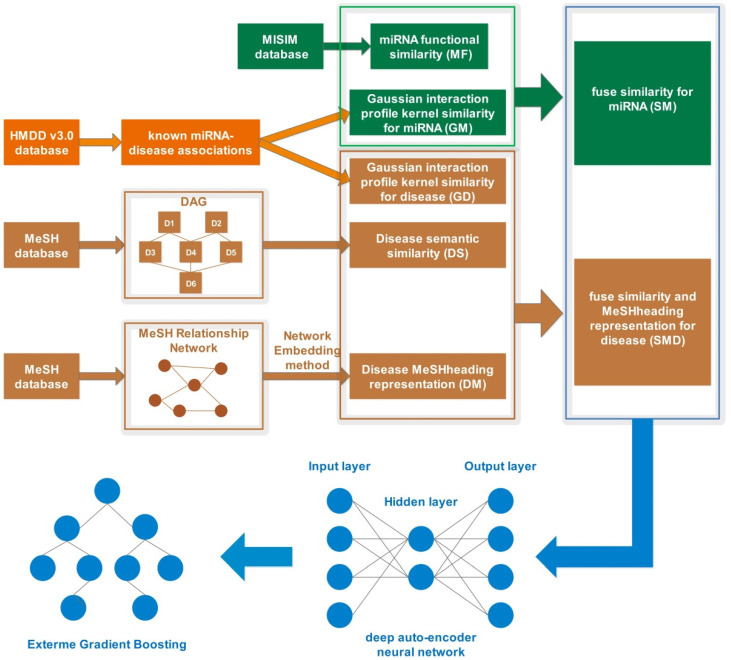
Flowchart of SMMDA to predict potential miRNA-disease associations.

In this work, we presented a novel computational method called SMMDA by incorporating multiple similarity profiles and a novel disease representation to accelerate the identification of potential miRNA-disease associations. The flowchart of SMMDA to predict potential miRNA-disease associations was shown in Figure 1. In summary, the main contributions of this paper are as follows below.

Considering the limited accessibility, high time consumption, and high cost of traditional biological research, a novel computational model called SMMDA was proposed to accelerate the identification of potential associations between miRNAs and diseases.

The multiple similarity profiles of miRNAs and diseases and a novel disease representative feature were incorporated to predict potential miRNA-disease associations, enhancing predictive accuracy.

Deep learning is used for high-quality extraction of integrated features, and the gradient boosting method is used for fast and highly accurate training and prediction.

Compared with previous related works, the experiment results have proved the superior performance of SMMDA for predicting potential miRNA-disease associations.

## 2. Materials and Methods

### 2.1. Human miRNA-Disease Associations

The HMDD v3.0 database (Human MicroRNA Disease Database) [19] contains 1102 miRNAs and 850 diseases and 32,281 associations in 17,412 papers. In our experiments, the positive dataset contains 1057 miRNAs, 850 diseases and 32,226 associations. What was removed were association data considered unreliable by the public database miRBase. In addition, we randomly selected 32,226 unrelated associations as the negative dataset, and it should be noted that these associations have been removed from the positive dataset.

### 2.2. miRNA Functional Similarity

Functional similarity between various miRNAs is a critical feature used for miRNA-disease association prediction, derived from the calculations of Wang et al. [20] They constructed a miRNA functional similarity score matrix (*MF*), available in http://www.cuilab.cn/files/images/cuilab/misim.zip (accessed on 1 March 2022), based on the principle that miRNAs with similar functions are more likely to be associated with diseases with similar phenotypes. Finally, the similarity score between miRNA $m_{1}$ and miRNA $m_{2}$ can be expressed as *MF(*$m_{1}$, $m_{2}$).

### 2.3. Gaussian Interaction Profile Kernel Similarity

Since miRNAs with similar functions are more likely to be associated with diseases with similar phenotypes and vice versa, we further calculated Gaussian interaction profile kernel similarity (GIP) for miRNAs and diseases [21]. In particular, an 850 rows and 1057 columns adjacency matrix was first constructed, with the rows in the matrix representing the number of miRNAs and the columns representing the number of diseases. The values of the elements in the matrix depend on whether there is an miRNA $m_{i}$ and disease $d_{j}$ association in the HMDD database; if it does, *MD*($m_{i}$, $d_{j}$) is equal to 1, otherwise it is equal to 0. The *i*-row vector of the adjacency matrix *MD* can be expressed as the binary vector *MD*($m_{i}$), denoting the interaction profiles of miRNA $m_{i}$. Based on the above definition, the GIP feature between miRNA $m_{i}$ and $m_{j}$, GM($m_{i}$, $m_{j}$), is defined as follows:

(1) $$\mathrm{GM}\left(m_{i},m_{j}\right)=\exp\left(-\delta_{m}{\|MD\left(m_{i}\right)-MD\left(m_{j})\right\|}^{2}\right)$$

where $\delta_{m}$ can be obtained by normalizing original parameter, which is the kernel bandwidth, as shown below:

(2) $$\delta_{m}=\frac{1}{m}\sum_{i=1}^{m}{\left\|MD\left(m_{i}\right)\right\|}^{2}$$

where *m* denotes the number of rows of the *MD*.

In the same way, the kernel similarity GD($d_{i}$, $d_{j}$) of the GIP similarity feature between disease $d_{i}$ and $d_{j}$ is defined as follow:

(3) $$\mathrm{GD}(d_{i},\;d_{j})=\exp(-\delta_{d}{\|MD\left(d_{i}\right)-MD\left(d_{j})\right\|}^{2})$$

(4) $$\delta_{d}=\frac{1}{d}\sum_{i=1}^{d}{\|MD\left(d_{i})\right\|}^{2}$$

where the total number of columns and *i*-column vector of the adjacent matrix *MD* are denoted by d and *MD*($d_{i}$).

### 2.4. Disease Semantic Similarity

The U.S. National Library of Medicine classifies all human diseases and has constructed the Medical Subject Headings (MeSH) database. According to this database division, we can use a directed acyclic graph (DAG) to represent each disease. For example, we can use DAG(D) = (D, *T*(D), E(D)) to represent a disease D, where *T*(D) denotes node D and all its ancestor nodes, and E(D) denotes the set of edges associated with node D. Further, we defined the contribution of node *d* in DAG(D) to the semantic value of disease node D as:

(5) $$\mathrm{DV}\left(D\right)=\sum_{d\in T\left(D\right)}D_{D}\left(d\right)$$

(6) $$\{\begin{array}{c} D_{D}\left(d\right)=1\;\mathrm{if}\;d=D\\ D_{D}\left(d\right)=\max\left\{\Delta_{*}D_{D}\left(d^{\prime }\right)|d^{\prime }\;\in \mathrm{children}\;\mathrm{of}\;d\right\}\;\mathrm{if}\;d\neq D \end{array}$$

where ∆ is the semantic contribution factor [20,22].

From the above equation, we can get that if two diseases have a larger shared part, then their similarity scores are higher. Therefore, the semantic similarity scores between diseases $d_{i}$ and $d_{j}$ are shown below:

(7) $$\mathrm{DS}(d_{i},\;d_{j})=\frac{\sum_{t\in T\left(d_{i}\right)\cap T\left(d_{j}\right)}\left(D_{d_{i}}\left(t\right)+D_{d_{j}}\left(t\right)\right)}{\mathrm{DV}\left(d_{i}\right)+\mathrm{DV}\left(d_{j}\right)}$$

### 2.5. MeSHHeading2vec Method

The characterization of diseases is an important part for predicting miRNA-disease associations, which is directly related to the prediction accuracy of the model. More and more researchers are focusing on high-quality feature representation of diseases, and in this section, we utilize a novel computational method, namely MeSHHeading2vec [23]. This new disease representation method compares to traditional GIP similarity features and semantic similarity features of diseases has been shown to have an even better performance. Specifically, a relational network is first constructed which transforms the MeSH tree structure of the diseases, connecting the different disease MeSH headings. In addition, the method calculates the node and edge number in the network and provides a brief analysis of the distribution of labels of nodes and the degree of distribution, where the pattern of tree numbers corresponding to a node determines the label (category) of each node (MeSH heading). Finally, different network representation learning methods including DeepWalk [24], LINE [25], SDNE [26], HOPE [27], and LAP [28] are applied to this relational network thus obtaining high-quality network features of the disease and retainning the raw node related information and network structure. Based on the method, the LINE network representation method was chosen for high-quality disease network feature extraction to enhance the predictive power of SMMDA for potential miRNA-disease associations

### 2.6. Incorporating Multiple Similarity Profiles and a Novel Disease Representation

In this section, multiple miRNA similarity profile features, disease similarity profile features, and new high-quality disease representation features are incorporating. Specifically, the final matrix MFM($m_{i}$*,* $m_{j}$) of miRNA feature is defined as follows:

(8) $$\mathrm{MFM}\left(m_{i},m_{j}\right)=\{\begin{array}{c} MF\left(m_{i},m_{j}\right),\;if\;m_{i}\;and\;m_{j}\;has\;functional\;similarity\;\\ \mathrm{GM}\left(m_{i},m_{j}\right),\;\;otherwise\; \end{array}$$

where GM denotes miRNA GIP similarity and *MF* denotes miRNA functional similarity matrix.

Similarly, the final disease feature matrix DFM($d_{i}$, $d_{j}$) is defined:

(9) $$\mathrm{DFM}(d_{i},\;d_{j})=\{\begin{array}{c} \mathrm{DM}\left(d_{i},\;d_{j}\right),\;\;\;if\;d_{i}\;and\;d_{j}\;has\;Meshheading\;feature\;\\ \mathrm{DS}\left(d_{i},\;d_{j}\right),\;\;if\;d_{i}\;and\;d_{j}\;has\;no\;Meshheading\;feature\\ \mathrm{GD}\left(d_{i},\;d_{j}\right),\;\;\;otherwise \end{array}$$

where DM denotes the new high-quality disease representation feature, DS denotes the disease semantic similarity feature and GD denotes the disease Gaussian interaction profile kernel similarity feature.

### 2.7. Deep Auto-Encoder Learning Method

For eliminating noise and reduce dimension of original features, the deep auto-encoder method (DAE) [29] was used for improving prediction accuracy of miRNA-disease associations in our work. Specifically, we constructed the deep learning framework containing 7 fully connected layers as hidden layers, where the number of neurons, respectively, is ($2^{9}$, $2^{8}$, $2^{7}$, $2^{6}$, $2^{7}$, $2^{8}$, $2^{9}$), and the activation function for each layer uses the ReLU function. The first 3 hidden layers are the encoding part, the last 3 hidden layers are the decoding part, and the output of the middle layer is the final reduced dimensional feature data. First, the encoding part projects the original features f from the input layer to the hidden layer *h*1 using the mapping function *y*1. Secondly, the decoding part projects the hidden part *h* to the output layer *h*2 by a mapping function *y*2.

(10) $$h1=y1\left(f\right)≔{\;S}_{y1}\left(\mathrm{Wf}+p\right)$$

(11) $$h2=y2\left(h1\right)≔{\;S}_{y2}\left(W\prime f+q\right)$$

Furthermore, the ReLU function is chosen as the activation function of AE in our work.

(12) $$S_{y1}\left(t\right)=S_{y2}\left(t\right)=\max\left(0,Wt+b\right)$$

### 2.8. Exterme Gradient Boosting

In recent years, the Exterme Gradient Boosting (XGBoost) proposed by Chen et al. is widely used by researchers and has yielded satisfactory results. XGBoost is a new classifier based on classification and regression trees integration (CART) and utilizes gradient boosting to optimize trees [30].

Set the output of a tree as shown below:

(13) $$F(x)=W_{q}\left(x_{i}\right)$$

where $W_{q}$ is the score of the leaf nodel q and $x_{i}$ is the input vector. On the basis, the output of the set of K trees is:

(14) $$y_{i}=\sum_{k=1}^{K}F_{k}\left(x_{i}\right)$$

The objective function O at step t of XGBoost method is:

(15) $$O(t)=\sum_{i=1}^{n}L\left(y_{i},{y^{\prime }}_{i}^{t-1}+{\;F}_{t}\left(x_{i}\right)\right)+\sum_{i=1}^{t}P\left(F_{i}\right)$$

where *L* is the train loss function between the output *y′* and real *y*, the second term in the function is for regularization.

Moreover, the complexity of the XGBoost method is defined as follows:

(16) $$P(F)=\gamma T+0.5\lambda \sum_{j=1}^{T}w_{j}^{2}$$

where γ is the pseudo-regularization hyperparameter, *T* is the total number of leaf nodes and λ is the L2 norm for leaf weights.

For detecting the optimal weights W, the gradient is used to conduct second-order approximation to the loss function, and the optimal value of the objective function is

(17) $$O(t)=-0.5*\sum_{j=1}^{T}{\left(\sum_{i\epsilon I}g_{i}\right)}^{2}*\;{\left(\sum_{i\epsilon I}h_{i}+\lambda \right)}^{-1}\;+\gamma \;T$$

where *I* is the set of leaf nodes, $g_{i}$ and $h_{i}$ are the gradient statistics on the loss function, given by:

(18) $$g_{i}=\partial_{y\prime^{t-1}}\;L(y_{i},{y^{\prime }}_{i}^{t-1})$$

(19) $$h_{i}=\partial^{2}{}_{y\prime^{t-1}}\;L(y_{i},{y^{\prime }}_{i}^{t-1})$$

## 3. Results and Discussion

### 3.1. The Detailed Prediction Performance of SMMDA

To accurately assess the predictive power of SMMDA for potential miRNA-disease associations, the more widely adopted five-fold cross-validation method was utilized. The method was repeated five times by randomly shuffling the samples and dividing them evenly into five parts, with one part as the test dataset and the remaining four groups as the training dataset. The detailed results of the experiments are recorded in Table 1, containing six commonly used predictive metrics, namely accuracy (Acc.), precision (Prec.), sensitivity (Sen.), Mathews correlation coefficient (MCC), and areas under the ROC curve (AUC). From the experimental results, we can see that SMMDA achieved a mean accuracy of 86.68% with a standard deviation of 0.42%, which is a good proof of the excellent performance of SMMDA. For the AUC metric, which is more indicative of the model’s predictive power, SMMDA obtained a mean of 94.06% with a standard deviation of 0.23% under five-fold cross-validation.

### 3.2. Comparison of Different Feature Combinations

To further assess the capability of our proposed feature descriptors, we compared them with different descriptors. In particular, the feature descriptors in our work is generated by fusing a novel disease representation, miRNA functional similarity, disease semantic similarity, and GIP kernel similarity information of miRNAs and diseases. Furthermore, a different feature descriptor is generated by only fusing miRNA functional similarity, disease semantic similarity, and GIP kernel similarity information of miRNAs and diseases (DescSim). The detailed results of the feature descriptors DescSim under 5-fold cross-validation were shown in Table 2. The results that our feature descriptors have a better performance than the feature descriptors used in many previous methods which only fuse similarity information to predict underlying miRNA-disease associations.

### 3.3. Comparison of Different Classifier Methods

**Figure 2:**
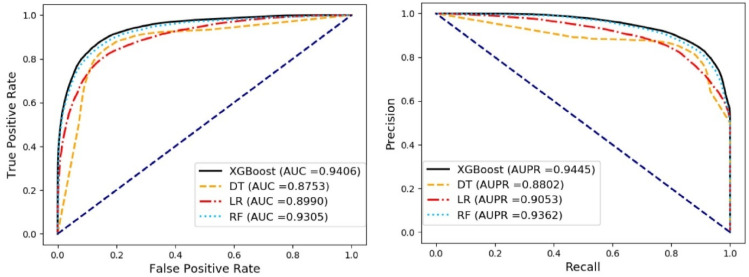
Comparison of SMMDA with random forest, logistic regression, decision tree and XGBoost classifiers.

In order to select the best predictive classifier method for SMMDA model, we conducted, respectively, the five-fold cross-validation experiment using different classifier methods including decision tree (DT) [31], logistic regression (LR) [32], random forest (RF) [33], and Extreme Gradient Boosting (XGBoost). It is worth noting that all experiments adopt the same environment and different classification methods adopt default training parameters to ensure the fairness and ease of operation of the comparison experiment. The average results of different classifier methods were displayed in Table 3. The AUC values and ROC curves, AUPR values and PR curves was respectively shown in the Figure 2. The comparison experiment demonstrates that XGBoost has a better performance than the other methods. Therefore, it is more suitable for SMMDA models.

### 3.4. Comparison of Previous Related Works

To further demonstrate the good performance of SMMDA, we compared 10 previous start-of-the-art computational models, namely DANE-MDA [16], MLMDA [34], MTDN [17], VAEMDA [18], LMTRDA [35], DBMDA [36], WBSMDA [37], PBMDA [38], HDMP [39], RLSMDA [40]. Furthermore, the data sets used by all these models are from the HMDD database. Here we selected the results of average AUC under five-fold cross-validation experiment as evaluation indicators. As shown in Table 4, SMMDA has a higher mean AUC value in the experiment, which proves its superior performance in the field of miRNA-disease association prediction.

### 3.5. Case Studies

To further evaluate whether SMMDA could perform accurately and robustly, we select three complex Human diseases for case studies including colon neoplasms, breast neoplasms, and esophageal neoplasms. Specifically, the known miRNA-disease associations in HMDD v3.0 [19] are selected as the training samples, and candidate miRNAs for evaluated diseases are ranked in compliance with the predictive scores provided by SMMDA. It is important to note that we have deleted the associations that have been verified in the HMDD v3.0 database to ensure that the validation data set is not correlated with the data set already used for training. Finally, we confirmed the top 50 predicted miRNA-disease associations with the dbDEMC [41] and miR2Disease [42] databases.

Colon neoplasms are cancers that begin in the final part of the digestive tract (colon). It can occur at any age, but the incidence is higher in the elder people. Colon neoplasms usually start as non-cancerous (benign) small cell clumps, called polyps, which form inside the colon. Overtime, a few polyps will become colon cancer. Hence, doctors recommend regular screening to identify and remove polyps before they become cancer, which can help prevent colon cancer. The SMMDA model was utilized to predict potential miRNA-esophageal-neoplasm associations. In the result, 47 of the top 50 predicted miRNAs are identified in the databases (see Table 5).

Breast neoplasms are cancers that occur in the breast cells. It is the most common cancer diagnosed in women in the United States, second only to skin cancer [43,44,45]. Breast neoplasms can occur in both men and women, but are much more severe in women. In recent years, the survival rates of breast neoplasms have increased largely due to factors such as a better understanding of the disease and earlier detection. In this article, SMMDA was utilized to predict potential miRNA-breast neoplasms associations. Finally, 48 of the top 50 predicted miRNAs are identified in the databases (see Table 6).

Esophageal Neoplasms are a serious digestive disease with a high death rate [46,47,48]. It is the sixth most common cause of cancer death worldwide. The incidence of it varies from place to place. In some areas, the higher incidence of esophageal neoplasms may be due to smoking and alcohol consumption or special nutritional habits and obesity [49,50]. In this article, SMMDA was utilized to predict potential miRNA-esophageal neoplasms associations. Finally, 48 of the top 50 predicted miRNAs are identified in the databases (see Table 7).

## 4. Conclusions

Recently, machine-learning approaches have been widely investigated in the field of bioinformatics including the prediction of potential associations between miRNAs and diseases. In this work, considering the limited accessibility, high time consumption and high cost in traditional biological researches, we presented a novel computational method called SMMDA by incorporating multiple similarity profiles and a novel disease representation to accelerate the identification of potential miRNA-disease associations. The multiple similarity profiles of miRNAs and diseases and a novel disease representative feature were incorporating, thereby enhancing predictive accuracy. The deep learning is used for high-quality extraction of integrated features and gradient boosting method is used for fast and highly accurate training and prediction. Compared with previous related works, the experiment results have proved that the superior performance of SMMDA. The comparison experiment of different classifiers and different feature descriptors further proved that the good predictive performance of SMMDA. In addition, the results of case studies with three Human diseases, including breast neoplasms, colon neoplasms, and esophageal neoplasms also demonstrated the feasibility of SMMDA in practical applications. Consequently, SMMDA was intended to be useful for the prediction of associations between miRNAs and diseases, and to be effective for prevention, diagnosis, treatment and prognosis of Human diseases.

## Figures and Tables

**Table 1:** The detailed prediction performance of SMMDA.

Fold	ACC. (%)	Spec. (%)	Sen.(%)	MCC (%)	Prec. (%)	AUC (%)
0	86.82	86.95	86.69	73.64	86.92	94.16
1	86.99	86.45	87.53	73.98	86.60	94.30
2	86.80	86.52	87.08	73.59	86.59	94.02
3	85.94	85.76	86.13	71.89	85.81	93.70
4	86.86	87.01	86.70	73.72	86.97	94.17
**Average**	**86.68 ± 0.42**	**86.54 ± 0.50**	**86.83 ± 0.52**	**73.36 ± 0.84**	**86.58 ± 0.46**	**94.06 ± 0.23**

**Table 2:** Evaluation of our method with different feature combinations.

Fold	ACC. (%)	Spec. (%)	Sen. (%)	MCC (%)	Prec. (%)	AUC (%)
0	86.64	86.61	86.67	73.29	86.62	94.15
1	86.58	86.10	87.06	73.16	86.23	94.10
2	86.32	86.41	86.24	72.65	86.38	93.68
3	87.02	86.72	87.32	74.04	86.80	94.07
4	86.45	86.10	86.81	72.91	86.20	93.84
**Average**	**86.60 ± 0.26**	**86.39 ± 0.29**	**86.82 ± 0.41**	**73.21 ± 0.52**	**86.45 ± 0.26**	**93.97 ± 0.20**
**SMMDA**	**86.68 ± 0.42**	**86.54 ± 0.50**	**86.83 ± 0.52**	**73.36 ± 0.84**	**86.58 ± 0.46**	**94.06 ± 0.23**

**Table 3:** Comparison of SMMDA with different classifier methods.

Classifier	ACC. (%)	Spec. (%)	Sen. (%)	MCC (%)	Prec. (%)	AUC (%)
DT	84.10 ± 0.15	83.30 ± 0.51	84.89 ± 0.33	68.20 ± 0.29	83.56 ± 0.38	87.53 ± 0.14
LR	82.50 ± 0.22	84.17 ± 0.66	80.82 ± 0.41	65.03 ± 0.45	83.62 ± 0.52	89.91 ± 0.21
RF	85.66 ± 0.36	85.61 ± 0.21	85.71 ± 0.63	71.32 ± 0.72	85.63 ± 0.22	93.05 ± 0.30
**XGBoost**	**86.68 ± 0.42**	**86.54 ± 0.50**	**86.83 ± 0.52**	**73.36 ± 0.84**	**86.58 ± 0.46**	**94.06 ± 0.23**

**Table 4:** Comparison of previous related works under the five-fold cross-validation.

Models	Average AUC (%)
DANE-MDA	92.64
MLMDA	91.72
MTDN	91.89
VAEMDA	90.91
LMTRDA	90.54
RLSMDA	85.69
PBMDA	91.72
WBSMDA	81.85
DBMDA	91.29
HDMP	83.42
**SMMDA**	**94.07**

**Table 5:** Top 50 potential colon neoplasms-related miRNAs, 47 were confirmed by dbDEMC and miR2Disease databases.

miRNA	Evidence	miRNA	Evidence
hsa-mir-122	dbDemc	hsa-mir-451	dbDemc; miR2Disease
hsa-mir-146b	dbDemc	hsa-mir-494	dbDemc
hsa-mir-34c	miR2Disease	hsa-mir-10a	dbDemc; miR2Disease
hsa-mir-375	dbDemc	hsa-mir-320a	dbDemc
hsa-mir-9	dbDemc	hsa-mir-19b	dbDemc; miR2Disease
hsa-mir-16	miR2Disease	hsa-mir-139	dbDemc; miR2Disease
hsa-mir-206	dbDemc; miR2Disease	hsa-mir-491	dbDemc
hsa-mir-1	dbDemc; miR2Disease	hsa-mir-26b	dbDemc
hsa-mir-183	dbDemc; miR2Disease	hsa-mir-212	dbDemc
hsa-mir-182	dbDemc; miR2Disease	hsa-mir-193b	dbDemc
hsa-mir-214	dbDemc; miR2Disease	hsa-mir-338	dbDemc
hsa-mir-27b	dbDemc; miR2Disease	hsa-mir-199a-2	miR2Disease
hsa-mir-34b	miR2Disease	hsa-mir-20b	dbDemc; miR2Disease
hsa-mir-26a	miR2Disease	hsa-mir-497	dbDemc; miR2Disease
hsa-mir-199a	miR2Disease	hsa-mir-129	miR2Disease
hsa-mir-429	dbDemc	hsa-mir-130b	dbDemc; miR2Disease
hsa-mir-29c	dbDemc; miR2Disease	hsa-mir-135a	dbDemc
hsa-mir-96	dbDemc; miR2Disease	hsa-mir-328	dbDemc; miR2Disease
hsa-mir-99a	dbDemc; miR2Disease	hsa-mir-503	dbDemc; miR2Disease
hsa-mir-100	dbDemc	hsa-mir-372	dbDemc; miR2Disease
hsa-mir-144	dbDemc	hsa-mir-133a-1	dbDemc
hsa-mir-483	Unconfirmed	hsa-mir-449b	dbDemc
hsa-mir-7	dbDemc; miR2Disease	hsa-mir-29	Unconfirmed
hsa-let-7	Unconfirmed	hsa-mir-98	dbDemc; miR2Disease
hsa-mir-196a-2	dbDemc; miR2Disease	hsa-mir-342	dbDemc; miR2Disease

**Table 6:** Top 50 potential breast neoplasms-related miRNAs, 48 were confirmed by dbDEMC and miR2Disease databases.

miRNA	Evidence	miRNA	Evidence
hsa-mir-95	dbDemc	hsa-mir-877	dbDemc
hsa-mir-99b	dbDemc; miR2Disease	hsa-mir-337	dbDemc
hsa-mir-190	dbDemc; miR2Disease	hsa-mir-138-1	miR2Disease
hsa-mir-217	dbDemc; miR2Disease	hsa-mir-650	dbDemc
hsa-mir-206	dbDemc; miR2Disease	hsa-mir-449b	dbDemc
hsa-mir-369	dbDemc	hsa-mir-550a	dbDemc
hsa-mir-19b-3p	dbDemc	hsa-mir-4717	Unconfirmed
hsa-mir-517a	dbDemc	hsa-mir-329	dbDemc
hsa-mir-422a	dbDemc	hsa-mir-639	dbDemc
hsa-mir-133	miR2Disease	hsa-mir-645	dbDemc
hsa-mir-4324	dbDemc	hsa-mir-1308	dbDemc
hsa-mir-378b	dbDemc	hsa-mir-572	dbDemc; miR2Disease
hsa-mir-431	dbDemc	hsa-mir-498	dbDemc; miR2Disease
hsa-mir-1908	dbDemc	hsa-mir-561	dbDemc; miR2Disease
hsa-mir-188	dbDemc	hsa-mir-1321	dbDemc
hsa-mir-658	dbDemc; miR2Disease	hsa-mir-154	dbDemc
hsa-mir-518e	dbDemc	hsa-mir-1825	dbDemc
hsa-mir-636	dbDemc	hsa-mir-504	dbDemc
hsa-mir-362	miR2Disease	hsa-mir-147b	dbDemc
hsa-mir-487b	dbDemc	hsa-mir-454	dbDemc
hsa-mir-501	dbDemc; miR2Disease	hsa-mir-208	dbDemc; miR2Disease
hsa-mir-665	dbDemc	hsa-mir-208b	dbDemc
hsa-mir-432	dbDemc	hsa-mir-1236	dbDemc
hsa-mir-30	Unconfirmed	hsa-mir-323	dbDemc
hsa-mir-511	dbDemc; miR2Disease	hsa-mir-186	dbDemc; miR2Disease

**Table 7:** Top 50 potential esophageal neoplasms-related miRNAs, 48 were confirmed by dbDEMC and miR2Disease databases.

miRNA	Evidence	miRNA	Evidence
hsa-mir-132	dbDemc	hsa-mir-195	dbDemc
hsa-mir-199a	dbDemc	hsa-mir-339	dbDemc
hsa-mir-29a	dbDemc	hsa-mir-18b	dbDemc
hsa-mir-19b	dbDemc	hsa-mir-101	dbDemc
hsa-mir-23b	dbDemc	hsa-mir-146b	dbDemc
hsa-mir-222	dbDemc	hsa-mir-196a	dbDemc; miR2Disease
hsa-mir-16	dbDemc	hsa-mir-103	dbDemc; miR2Disease
hsa-mir-29b	dbDemc	hsa-mir-215	dbDemc
hsa-mir-429	dbDemc	hsa-mir-224	dbDemc
hsa-mir-182	dbDemc	hsa-mir-137	Unconfirmed
hsa-mir-125a	dbDemc	hsa-mir-24	dbDemc
hsa-mir-181b	dbDemc	hsa-mir-335	dbDemc
hsa-mir-499	dbDemc	hsa-mir-144	dbDemc
hsa-mir-7	dbDemc	hsa-mir-15b	dbDemc
hsa-let-7i	dbDemc	hsa-mir-497	dbDemc
hsa-mir-133a	dbDemc	hsa-mir-106a	dbDemc
hsa-mir-20b	dbDemc	hsa-mir-26a	dbDemc
hsa-mir-221	dbDemc	hsa-mir-218	dbDemc
hsa-mir-204	dbDemc	hsa-let-7f	dbDemc
hsa-mir-181a	dbDemc	hsa-mir-139	dbDemc
hsa-mir-302c	Unconfirmed	hsa-mir-124	dbDemc
hsa-mir-378	dbDemc	hsa-mir-206	Unconfirmed
hsa-mir-1	dbDemc	hsa-mir-372	dbDemc
hsa-mir-18a	dbDemc	hsa-mir-23a	Unconfirmed
hsa-mir-199b	dbDemc	hsa-mir-10a	dbDemc

## Data Availability

The datasets analyzed during the current study are available from the cor-responding author on reasonable request.
